# Gestational valproic acid exposure enhances facial stimulation-evoked cerebellar mossy fiber-granule cell transmission via GluN2A subunit-containing NMDA receptor in offspring mice

**DOI:** 10.1038/s41398-024-02990-0

**Published:** 2024-07-03

**Authors:** Yong-Xue Yuan, Yang Liu, Jing Zhang, Yan-Hua Bing, Chao-Yue Chen, Guang-Gao Li, Chun-Ping Chu, Ming-Ji Yin, De-Lai Qiu

**Affiliations:** 1 Department of Physiology and Pathophysiology, College of Medicine, Yanbian University, Yanji City, 133002 Jilin China; 2 Department of Orthopedics, Affiliated Hospital of Yanbian University, Yanji City, 133000 Jilin China; 3 Institute of Brain Science, Jilin Medical University, Jilin City, 132013 Jilin China; 4 Department of Physiology, College of Basic Medicine, Jilin Medical University, Jilin City, 132013 Jilin China; 5 Functional Experiment Center, College of Medicine, Yanbian University, Yanji City, 133000 Jilin China; 6 Department of Pediatrics, Affiliated Hospital of Yanbian University, Yanji City, 133000 Jilin China

**Keywords:** Autism spectrum disorders, Neuroscience

## Abstract

Valproic acid (VPA) is one of the most effective antiepileptic drugs, and exposing animals to VPA during gestation has been used as a model for autism spectrum disorder (ASD). Numerous studies have shown that impaired synaptic transmission in the cerebellar cortical circuits is one of the reasons for the social deficits and repetitive behavior seen in ASD. In this study, we investigated the effect of VPA exposure during pregnancy on tactile stimulation-evoked cerebellar mossy fiber-granule cell (MF-GC) synaptic transmission in mice anesthetized with urethane. Three-chamber testing showed that mice exposed to VPA mice exhibited a significant reduction in social interaction compared with the control group. In vivo electrophysiological recordings revealed that a pair of air-puff stimulation on ipsilateral whisker pad evoked MF-GC synaptic transmission, N1, and N2. The evoked MF-GC synaptic responses in VPA-exposed mice exhibited a significant increase in the area under the curve (AUC) of N1 and the amplitude and AUC of N2 compared with untreated mice. Cerebellar surface application of the selective N-methyl-D-aspartate (NMDA) receptor blocker D-APV significantly inhibited facial stimulation-evoked MF-GC synaptic transmission. In the presence of D-APV, there were no significant differences between the AUC of N1 and the amplitude and AUC of N2 in the VPA-exposed mice and those of the untreated mice. Notably, blockade of the GluN2A subunit-containing, but not the GluN2B subunit-containing, NMDA receptor, significantly inhibited MF-GC synaptic transmission and decreased the AUC of N1 and the amplitude and AUC of N2 in VPA-exposed mice to levels similar to those seen in untreated mice. In addition, the GluN2A subunit-containing NMDA receptor was expressed at higher levels in the GC layer of VPA-treated mice than in control mice. These results indicate that gestational VPA exposure in mice produces ASD-like behaviors, accompanied by increased cerebellar MF-GC synaptic transmission and an increase in GluN2A subunit-containing NMDA receptor expression in the offspring.

## Introduction

Autism spectrum disorder (ASD) is a group of neurodevelopmental disorders that primarily affect social cognition and manifest as social, including poor or no language development, enhanced sensory sensitivity, repetitive behaviors, attention abnormalities, and resistance to novel environments [1]. ASD can be caused by various factors, such as genetic and environmental influences. An earlier study showed that ASD can also include motor dysfunction, such as visual motor and flexibility disorders, limb coordination disorders that affect balance, flexibility, and speed, as well as gait disorders and ataxia [2]. The cellular and molecular mechanisms underlying ASD symptoms are thought to be related to alterations in synaptic transmission and neuronal circuit function, including excitatory and inhibitory synaptic transmission [3–6].

Valproic acid (VPA) is frequently used to treat both epilepsy and nonepileptic diseases [7–9]. However, prenatal exposure to VPA has several well-known side effects, such as neural tube defects, facial abnormalities, reduced intelligence, and high risk of ASD [10–14]. There are several known mechanisms by which VPA enhances the risk of ASD, such as increased acetylation of histone proteins [15–18] and altered synaptic development, transmission, and long-term plasticity [19–26].

Prenatal VPA exposure enhances N-metyl-D-aspartate (NMDA) receptor-mediated synaptic transmission and plasticity, which may cause ASD-like behaviors [19]. Prenatal exposure to VPA in rats enhances NMDA receptor function by increasing expression of the NMDA receptor subunits GluN2A and GluN2B in the rat brain [19]. Behavioral experiments have shown that pharmacological inhibition of NMDA receptor function improves social deficits in rats exposed to VPA [24] and improves social deficits and repetitive behaviors in VPA-exposed mice [27]. NMDARs are found on the membrane of cerebellar granule cells (GCs) and parallel fiber boutons, which play critical roles in sensory information processing, synaptic plasticity, motor learning and memory, neuropathy, and cerebellar disorders [28–30]. In rats, GluN2A and GluN2C mRNA expression can be detected in cerebellar GCs during the second postnatal week, whereas GluN2B mRNA is expressed transiently in GCs during the first 2 postnatal weeks [31]. Later studies showed that GluN1 and GluN2 are expressed in GC somas and parallel fiber boutons [28, 29], while the GluN2A and GluN2C subunits are expressed on the postsynaptic membranes of MF-GC synapses in the adult mouse cerebellum [32]. We recently showed that NMDA receptors, especially GluN2A, contribute to facial stimulation-evoked MF-GC synaptic transmission, indicating that NMDA receptors play a critical role in lateral sensory information synaptic transmission in the mouse cerebellar granular layer [33]. Prenatal exposure to VPA enhances NMDA receptor function in the rat brain [19], and pharmacological inhibition of NMDA receptor function improves social deficits and repetitive behaviors in VPA-exposed mice [24, 27], suggesting that abnormal NMDA receptor function in cerebellar GCs affecting MF-GC synaptic transmission may occur in ASD model mice.

Recent evidence suggests that cerebellar circuit function may contribute to the motor impairment seen in ASD [34, 35]. Cerebellar abnormalities, especially in Crus I/II lobules, have been consistently demonstrated in patients with ASD [36, 37]. Reduced connectivity between the right Crus I/II and the prefrontal regions correlates with increasing ASD symptom severity [38, 39], and disrupted right Crus I-cerebral cortex connectivity is evident in the Purkinje cell Tsc1-mutant mouse model of ASD [40, 41], indicating the importance of the Crus I/II lobules in the control of social behaviors and their altered functions in ASD. ASD model mice exhibit both motor and social deficits, which correlate with a loss of Purkinje cells within the Crus I lobule of the cerebellar cortex [40, 41]. In contrast, air-puff stimulation of the ipsilateral whisker pad evokes strong synaptic responses in the Crus II lobule of the cerebellar cortex through the MF pathway in mice [42, 43], suggesting that the Crus II lobule is also critical for regulating social behavior in mice. Moreover, the cerebellar flocculus complex contributes to the hypersensitized vestibulo-ocular reflex seen in ASD model mice, suggesting that aberrant cerebellar circuit function may mediate abnormally strong sensory responses [44]. Although abnormalities in the Crus I lobule are closely related to ASD, whether abnormal sensory information transmission occurs in the Crus II lobule of the mouse cerebellar cortex is unknown. Therefore, in this study we used a VPA-exposed mouse model of ASD to investigate the effect of prenatal exposure to VPA on facial stimulation-evoked MF-GC synaptic transmission in the Crus II lobule of the cerebellar cortex.

## Methods

### Prenatal exposure to VPA

Adult C57BL/6J mice were mated and examined for the presence of a vaginal plug, which was employed to define embryonic day 0 (E0). Pregnant mice (*n* = 10 in each group) received a single subcutaneous injection of sodium valproic (Sigma) in saline (VPA; 600 mg/kg) or saline alone (control) at embryonic day 13.5. Male offspring from VPA-exposed and control group were retained and randomly used to perform further experiments. All behavioral and electrophysiological experiments were performed on 8- to 12-week-old offspring of these pregnant mice. All experimental procedures were approved by the Animal Care and Use Committee of Yanbian University (permit number SYXK (Ji) 2011-006) and were performed in accordance with the animal welfare guidelines of the U.S. National Institutes of Health, and the Animal Research: Reporting in Vivo Experiments (ARRIVE; https://arriveguidelines.org). All animals were housed under a 12-h light/12-h dark cycle with free access to food and water in a colony room maintained at a constant temperature (24 ± 1 °C) and humidity (50 ± 5%).

### Behavioral testing

ASD is characterized by deficits in social communication and stereotyped or repeated behaviors [23, 27, 45, 46]. We used the three-chamber social test to assess social interaction and the open field test to evaluate repetitive behaviors and exploration of novel environments. The three-chamber test was described previously [45]. The apparatus consists of a central chamber and two side chambers (40 × 20 × 22 cm). Each test included three 10-min sessions. First, the mouse was acclimated to the center chamber. Second, the mouse was allowed to explore all three chambers. Third, an unfamiliar mouse in a small plastic cage was placed in the left or right chamber (chosen randomly to avoid side preference), and the original mouse was allowed to explore all three chambers and the cage. The unfamiliar mouse was habituated to the plastic cage in the three-chamber apparatus for 30 min 24 h before the test. The amount of time spent in each chamber and sniffing the cage containing the strange mouse was measured using Smart 3.0 software (Panlab, Harward Apparatus). The preference index (%) was calculated using the formula (S − E)/(S + E) × 100, where S and E denote the stranger cage and empty cage, respectively [27].

The open field test was performed to evaluate exploratory and repetitive behaviors in mice. The total time spent on self-grooming, the time spent in the central area (the middle square of the nine-square grid), and the total distance traveled during the 5-min test were recorded for each mouse. The results from the three-chamber test and open field test were measured and analyzed using Smart 3.0 software (Panlab, Harward Apparatus). Mice that had been exposed to VPA prenatally spent significantly less time in the central zone, more time on self-grooming, less time sniffing the cage containing an unfamiliar mouse, and a smaller preference index compared with control group, all of which are identified as ASD-like behaviors [23, 27, 45, 46].

### In vivo electrophysiological recordings

The anesthesia and surgical procedures performed in this study have been described previously [33, 47]. The mice were anesthetized with urethane (1.3 mg/kg body weight, i.p.) and tracheotomized to avoid respiratory obstruction. A 1- to 1.5-mm hole was drilled in the skull to expose the cerebellar surface corresponding to Crus II. The cerebellar surface was superfused with oxygenated artificial cerebrospinal fluid (ACSF: 125 mM NaCl, 3 mM KCl, 1 mM MgSO_4_, 2 mM CaCl_2_, 1 mM NaH_2_PO_4_, 25 mM NaHCO_3_, and 10 mM d-glucose) with a peristaltic pump (Gilson Minipulse 3; Villiers, Le Bel, France) at 0.5 ml/min. Rectal temperature was monitored and maintained at 37.0 ± 0.2 °C.

Extracellular recordings of the cerebellar granular layer were performed with an Axopatch-200B amplifier (Molecular Devices, Foster City, CA, USA). The potentials were acquired through a Digidata 1440 series analog-to-digital interface on a personal computer using Clampex 10.4 software (Molecular Devices). The recording electrodes were filled with ACSF and had resistances of 3–5 MΩ. To record the facial stimulation-evoked MF-GC synaptic responses, a recording electrode was placed in the GL at a depth of 300–350 μm under the pia mater membrane [48].

Facial stimulation was performed by delivering an air puff to the ipsilateral whisker pad through a 12-gauge stainless steel tube connected to a pressurized injection system (Picospritzer^®^ III; Parker Hannifin Co., Pine Brook, NJ, USA). The air-puff stimuli, controlled by a personal computer, were synchronized with the electrophysiological recordings and delivered at 0.05 Hz via a Master 8 controller (A.M.P.I., Jerusalem, Israel) and Clampex10.4 software. To isolate MF-GC synaptic transmission, picrotoxin (100 µM) was added to the ACSF during all recordings to prevent GABA_A_ receptor-mediated inhibition. In the presence of picrotoxin, a pair of air-puff stimuli (10 ms, 60 psi) evoked double negative N1 and N2 components in the GL of the cerebellar cortical folium Crus II. On the basis of our previous studies [47, 48], N1 and N2 were identified as MF-GC synaptic transmissions evoked by the first and second stimuli, respectively. Picrotoxin, sodium valproate, and D-(-)-2-Amino-5-phosphonopentanoic Acid (D-APV) were purchased from Sigma-Aldrich (Shanghai, China). [(S)-[[(1S)-1-(4-bromophenyl)ethyl]amino]-(2,3-dioxo-1,4-dihydroquinoxalin -5-yl) methyl] phosphonic acid (PEAQX) and 2-((4-(2-fluorobenzyl)piperidin-1-yl)methyl)-1H-benzo[d]imidazol-6-ol dihydrochloride (TCN 237) were purchased from Tocris (Bristol, UK). The drugs were dissolved in ACSF and applied directly to the cerebellar surface using a peristaltic pump (0.5 ml/min).

### Immunohistochemistry and imaging

Mice (*n* = 6) were deeply anesthetized by intraperitoneal injection with chloral hydrate (7%; 5 ml/kg) and then transcardially perfused with cold phosphate buffer (PBS; pH 7.4), followed by 4% paraformaldehyde (PFA, Sinopharm Chemical Reagent Co, China) in PBS. The brains were harvested and post-fixed in PFA for 48 h at 4 °C, then washed with PBS. Next, the cerebellum was separated from the brain with a razor blade and soaked in a sucrose/PBS solution for at least 6 h. After embedding in Tissue-Tek O.C.T. Compound (Beijing Zhong Shan Jin Qiao Biotechnology Co, China), the cerebellum was quickly frozen at −80 °C for 2 h. Then, the cerebellum was sectioned into 8-μm-thick sagittal slices using a freezing microtome (CM1900, Leica, Germany). The sections were thawed at 25 °C for 30 min, fixed with 4 °C precooled acetone, and stored at 4 °C for immunohistochemical experiments. Cerebellar sections mounted on microscope slides were permeabilized with 0.3% Triton X-100 in PBS, blocked with 10% donkey serum in PBS, and incubated with rabbit anti-GluN2A (1:50, Abcam) overnight at 4 °C, followed by incubation with Alexa Fluor 488 donkey anti-rabbit (1:1000, ThermoFisher) and 4′,6-diamidino-2-phenylindole (DAPI, 1:1000) for 2 h at room temperature. Fluorescence images were acquired using a confocal laser-scanning microscope (Nikon C2, Tokyo, Japan) [37].

The electrophysiological data were analyzed using Clampfit 10.4 software (Molecular Devices, Foster City, CA, USA). ImageJ software (V1.8.0) was used to analyze the average fluorescence intensity of GluN2A in the granular cell layer. The mean gray value was calculated as follows: Integrate fluorescence intensity of the region/Area of the region. All data are expressed as the mean ±S.E.M. Differences between the mean values recorded under baseline conditions (ACSF) and test conditions were evaluated by paired Student’s *t* test, whereas the mean values of VPA-exposed mice and untreated mice were compared by one-way ANOVA (SPSS software; Chicago, IL). *P*-values below 0.05 were considered to be statistically significant.

## Results

### Mice exposed to VPA in utero exhibit impaired social interaction

**Fig. 1:**
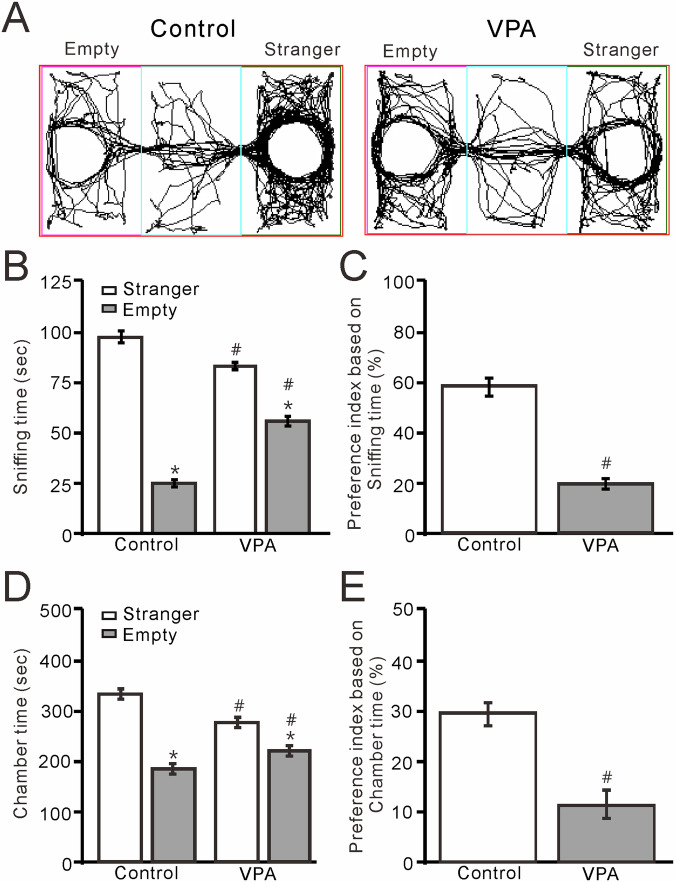
VPA-exposed mice exhibit ASD-like social interaction in the three-chamber test. **A** Representative traces showing the movements of control (unexposed) and VPA-exposed mice during the social interaction three-chamber test. **B** Time spent sniffing a cage containing a strange mouse or an empty cage by control and VPA-exposed mice. **C** Bar graph showing the preference index based on sniffing time, as shown in **B**. **D** Pooled data showing the time spent in the chamber with the strange mouse or an empty cage by control and VPA-exposed mice. **E** Bar graph showing the preference index based on time spent in the chamber containing the strange mouse or an empty cage, as shown in **D**. *n* = 10 mice in each group. *P < 0.05, strange mouse versus empty; #*P* < 0.05, V*P*A versus control.

**Fig. 2:**
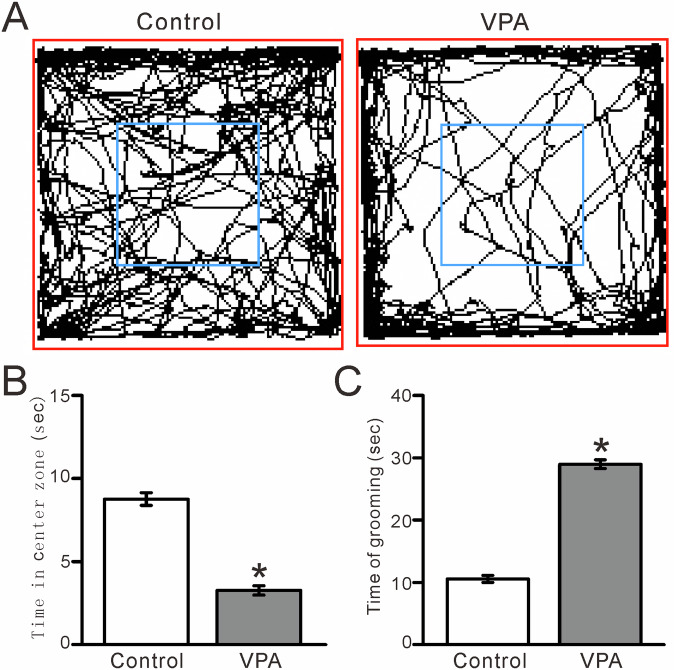
VPA-exposed mice exhibit ASD-like repetitive behaviors in the open field test. **A** Representative traces showing the movements of control (unexposed) and VPA-exposed mice during the 5-min open field test. **B** Bar graph showing the time spent in the central zone in the open field test. **C** Pooled data showing the time spent self-grooming in the open-field test. *n* = 7 mice in each group. *P < 0.05, VPA versus control.

To determine whether prenatal exposure to VPA impairs social interactions, we performed the three-chamber test to assess the relative preference of 8- to 12-week-old mice for exploring a cage containing a strange (S) mouse versus an empty (E) cage. We found that VPA exposure resulted in a significant reduction in social interaction compared with unexposed (control) mice. The amount of time that VPA-exposed mice spent sniffing the cage containing an unfamiliar mouse was significantly reduced compared with unexposed mice (control) (*P* < 0.01, *n* = 10; Fig. 1A), and the preference index based on sniffing time was significantly smaller in VPA-exposed mice than in control mice (*P* < 0.01, *n* = 10; Fig. 1C). The amount of time that the VPA-exposed mice spent in the cage containing the strange mouse was significantly reduced compared with unexposed mice (*P* < 0.01, *n* = 10; Fig. 1D), and the preference index based on time spent in the chamber was significantly smaller in the VPA-exposed mice than in the control mice (*P* < 0.01, *n* = 10; Fig. 1E). These results indicated that prenatal exposure to VPA causes deficits in - social interaction later in life. The open field experiments showed that VPA-exposed mice spent significantly less time in the central zone (*P* < 0.0001, *n* = 7; Fig. 2A, B) and more time on self-grooming (*P* < 0.0001, *n* = 7; Fig. 2C) than control mice, indicating that VPA exposure increased repetitive behaviors and reduced exploration of novel environments.

### Properties of facial stimulation-evoked mossy fiber-granular cell (MF-GC) synaptic transmission in VPA-exposed mice

**Fig. 3:**
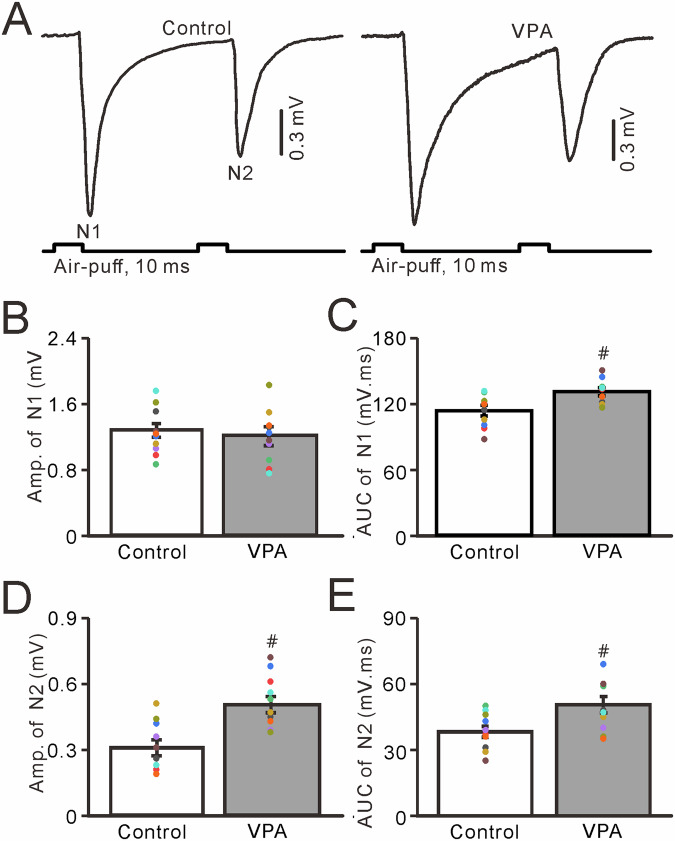
Properties of the cerebellar MF-GC synaptic transmission evoked by facial stimulation in VPA-exposed mice. **A** Representative traces showing that the paired air-puff stimulation (10 ms, 60 psi) of the ipsilateral whisker pad evoked MF-GC synaptic transmission in control and VPA-exposed mice. Bar graphs showing the amplitude (**B**) and area under the curve (**C**) of N1. **D**, **E** Amplitude (**B**) and area under the curve (**C**) of N2. Facial stimulation-evoked MF-GC synaptic transmission was significantly greater in VPA-exposed mice than in control mice. *n* = 10 mice in each group. # *P* < 0.05, VPA versus control.

In the presence of a GABA_A_ receptor antagonist, paired air-puff stimulation of the ipsilateral whisker pad (10 ms, 50–60 psi, 50-ms interval) evoked a few negative N1 and N2 components in the cerebellar granular layer, which were identified as facial stimulation-evoked MF-GC synaptic transmission (Fig. 3A) [33, 47–49]. The mean amplitude of N1 in VPA-exposed mice did not differ significantly from that in untreated mice (*P* > 0.05, *n* = 10; Fig. 3B), whereas the mean area under the curve (AUC) of N1 in VPA-exposed mice was significantly larger than that in untreated mice (*P* < 0.05, *n* = 10; Fig. 3C). Notably, facial stimulation evoked a stronger N2 in VPA-exposed mice than in untreated mice. The mean amplitude of the facial stimulation-evoked N2 in VPA-exposed mice was significantly higher than that in untreated mice (*P* < 0.01, *n* = 10; Fig. 3D), and the mean AUC of N2 in VPA-exposed mice was significantly larger than that in untreated mice (*P* < 0.01, *n* = 10; Fig. 3E). These results indicate that VPA exposure significantly enhances facial stimulation-induced MF-GC synaptic transmission in mice.

### Prenatal VPA exposure enhances MF-GC synaptic transmission via NMDA receptors

**Fig. 4:**
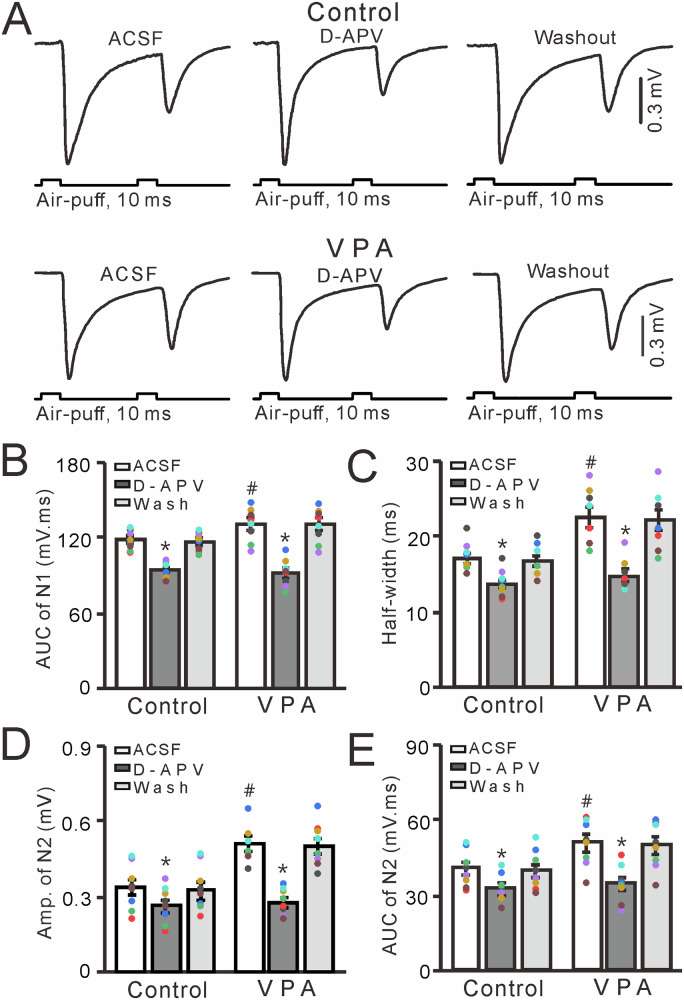
Effect of the NMDAR blocker D-APV on facial stimulation-evoked MF-GC synaptic transmission in VPA-exposed mice. **A** Representative traces showing that paired air-puff stimuli (10 ms, 60 psi) of the ipsilateral whisker pad evoked MF-GC synaptic transmission in control and VPA-exposed mice treated with ACSF, D-APV (250 μM), and subjected to washout. Mean (±S.E.M.) of the amplitude (**B**) and half-width (**C**) of N1in mice treated with ACSF or D-APV or subjected to washout. Mean value (±S.E.M) of the amplitude (**D**) and area under the curve (**E**) of N2 in mice treated with ACSF or D-APV or subjected to washout. *n* = 8 in each group. *P < 0.05, D-APV versus ACSF; #*P* < 0.05, VPA versus control.

NMDA receptors contribute to facial stimulation-evoked MF-GC synaptic transmission in the mouse cerebellar cortex [33, 47, 49], and therefore we further examined the effect of a selective NMDAR antagonist, D-APV, on facial stimulation-evoked MF-GC synaptic transmission in VPA-exposed mice and untreated mice. Treatment with D-APV (250 μM) significantly inhibited facial stimulation-evoked MF-GC synaptic transmission in both control (untreated) and VPA-exposed mice (*P* < 0.001 vs. ACSF; Fig. 4A). In the presence of D-APV, the mean AUC and half-width of N1 were significantly lower than those observed in control and VPA-exposed mice treated with ACSF alone (*P* < 0.001, *n* = 8; Fig. 4B, C). Notably, the mean AUC of N1 in VPA-exposed mice treated with ACSF was significantly larger than that observed in the control group treated with ACSF (*P* < 0.05, *n* = 8), but the N1 AUCs were similar in both groups treated with D-APV (*P* > 0.05, *n* = 8; Fig. 4B, C). In the presence of D-APV, the mean value of amplitude and AUC of N2 were significantly lower than those seen in control and VPA-exposed mice treated with ACSF (*P* < 0.001, *n* = 8; Fig. 4D, E). Furthermore, the mean value of amplitude and AUC of N2 were significantly larger in VPA-exposed mice treated with ACSF than in control mice treated with ACSF (*P* < 0.05, *n* = 8), but were similar in both groups treated with D-APV (*P* > 0.05, *n* = 8; Fig. 4D, E). These results indicate that NMDA receptor blockade inhibits MF-GC synaptic transmission and abolishes the increase in facial stimulation-evoked MF-GC synaptic transmission seen in VPA-exposed mice.

### Enhancement of MF-GC synaptic transmission in VPA-exposed mice via the GluN2A subunit-containing NMDA receptor

**Fig. 5:**
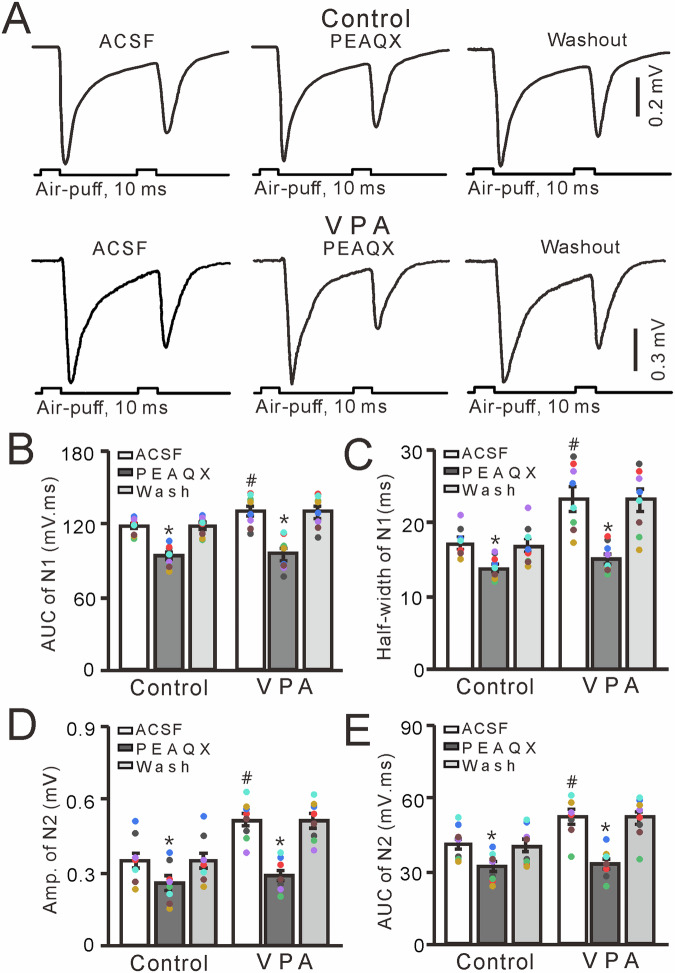
Effect of a selective GluN2A-containing NMDA receptor antagonist PEAQX (10 μM) on facial stimulation-evoked MF-GC synaptic transmission in VPA-exposed mice. **A** Representative traces showing that paired air-puff stimulation (10 ms, 60 psi) of the ipsilateral whisker pad evoked MF-GC synaptic transmission in control and VPA mice treated with ACSF or PEAQX (10 μM) or subjected to washout. Mean (±S.E.M) of the amplitude (**B**) and half-width (**C**) of N1 in mice treated with ACSF or PEAQX or subjected to washout. Mean (±S.E.M) of the amplitude (**D**) and area under the curve (**E**) of N2 in mice treated with ACSF or PEAQX or subjected to washout. *n* = 8 in each group. *P < 0.05, PEAQX versus ACSF; #*P* < 0.05, VPA versus control.

In the cerebellar cortex, GluN2A is expressed on the somas of GCs and the boutons of their axons [28, 29] and contributes to facial stimulation-evoked MF-GC synaptic transmission in mice [33, 47, 49]. Therefore, we next used a GluN2A-containing NMDA receptor antagonist, PEAQX (10 µM), to determine whether the increase in facial stimulation-evoked MF-GC synaptic transmission was mediated by the GluN2A-containing NMDA receptor. Similar to the results obtained by treatment with D-APV, cerebellar surface perfusion with PEAQX (10 μM) significantly inhibited facial stimulation-evoked MF-GC synaptic transmission in both control (VPA-untreated) and VPA-exposed mice (*P* < 0.001 vs. ACSF; Fig. 5A). The mean AUC and half-width of N1 were significantly lower in control and VPA-exposed mice treated with PEAQX than those in both groups treated with ACSF (*P* < 0.001, *n* = 8; Fig. 5B, C). The mean AUC of N1 in VPA-exposed mice treated with ACSF was significantly larger than that in control mice (*P* < 0.05, *n* = 8), but the mean AUC of N1 of both groups treated with PEAQX were similar (*P* > 0.05, *n* = 8; Fig. 5B, C). The mean amplitude and AUC of N2 were significantly lower in control and VPA-exposed mice treated with PEAQX than in both groups treated with ACSF (*P* < 0.001, *n* = 8; Fig. 5D, E). In addition, these values were significantly larger in VPA-exposed mice treated with ACSF than in control mice treated with ACSF (*P* < 0.05, *n* = 8), but were similar in both groups treated with PEAQX (*P* > 0.05, *n* = 8; Fig. 5D, E). These results indicate that GluN2A-containing NMDA receptor blockade inhibits MF-GC synaptic transmission and abolishes the increase in facial stimulation-evoked MF-GC synaptic transmission in VPA-exposed mice.

**Fig. 6:**
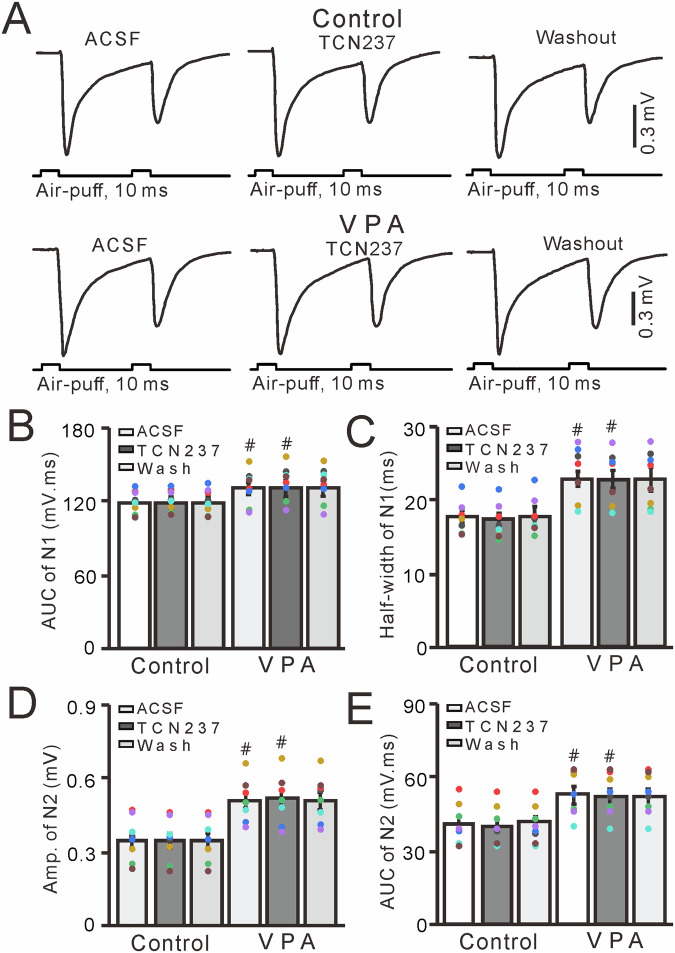
GluN2B-containing NMDA receptor blockade does not alter facial stimulation-evoked MF-GC synaptic transmission in VPA-exposed mice. **A** Representative traces showing that paired air-puff stimulation (10 ms, 60 psi) of the ipsilateral whisker pad evoked MF-GC synaptic transmission in control and VPA-exposed mice treated with ACSF or TCN (10 μM) or subjected to washout. Mean (±S.E.M) of the amplitude (**B**) and half-width (**C**) of N1 in mice treated with ACSF or TCN or subjected to washout. Mean (±S.E.M) of the amplitude (**D**) and area under the curve (**E**) of N2 in mice treated with ACSF or TCN or subjected to washout. *n* = 8 in each group. #*P* < 0.05, VPA versus control.

Next, we used the selective GluN2B antagonist TCN237 (10 µM) to determine whether the increase in facial stimulation-evoked MF-GC synaptic transmission observed in VPA-exposed mice was dependent on GluN2B-containing NMDA receptors [33, 47, 49]. Cerebellar surface perfusion with TCN237 did not significantly change facial stimulation–evoked MF-GC synaptic transmission in either control (untreated) or VPA-exposed mice (*P* > 0.05 vs. ACSF; Fig. 6A). In the presence of TCN237, the mean AUC and half-width of N1 were not significantly different from those seen in control and VPA-exposed mice treated with ACSF (*P* > 0.05, *n* = 8; Fig. 6B, C), but these values were significantly higher in VPA-exposed mice treated with TCN237 than in control mice treated with TCN237 (*P* < 0.05, *n* = 8; Fig. 6B, C). There was no significant difference in the mean amplitude and AUC of N2 in control and VPA-exposed mice treated with ACSF (*P* > 0.001, *n* = 8; Fig. 6D, E). However, both of these values were significantly higher in VPA-exposed mice treated with ACSF or TCN237 compared with control mice treated with ACSF or TCN237 (*P* < 0.05, *n* = 8; Fig. 6D, E). These results indicate that GluN2B-containing NMDA receptor blockade does not prevent the increase in facial stimulation-evoked MF-GC synaptic transmission in VPA-exposed mice.

**Fig. 7:**
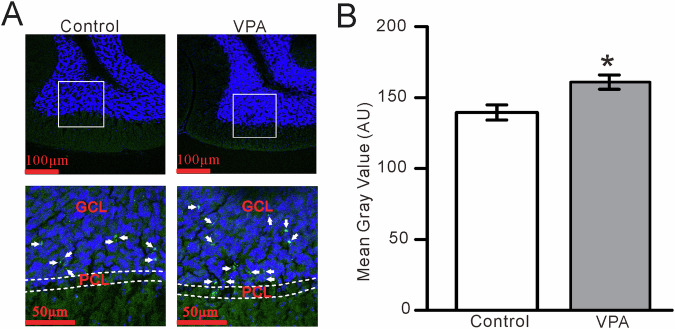
GluN2A subunit-containing NMDA receptor expression in the cerebellar GC layer is increased in VPA-exposed mice compared with control mice. **A** (upper panel) Digital micrographs showing confocal images of the cerebellar Crus II lobule of control (left) and VPA-exposed (right) mice. The nuclei are stained with DAPI (blue). (Lower panel) Higher magnifications of the boxed areas in (Upper panel) showing GluN2A subunit-containing NMDA receptor immunoreactivity in the GL (Green; arrows). PCL Purkinje cell layer, GCL granule cell layer. **B** Mean gray values of GluN2A subunit immunoreactivity in the cerebellar Crus II lobule in control and VPA-exposed mice. *P < 0.05, VPA versus control.

Next, we investigated GluN2A-containing NMDA receptor expression in the cerebellar granular layer of VPA-exposed mice by confocal laser-scanning microscope. The GluN2A subunit-containing NMDA receptor was expressed in the GC layer of both VPA-treated and control mice (Fig. 7A), but at significantly higher levels in VPA-treated mice (Fig. 7B). Collectively, these results indicate that an increase in GluN2A-containing NMDA receptor expression in the cerebellar GC layer of VPA-exposed mice increased facial stimulation-evoked MF-GC synaptic transmission.

## Discussion

The main finding from this study is that mice exposed to VPA in utero exhibited significantly greater sensory stimulation-evoked MF-GC synaptic transmission than unexposed mice. This increase in MF-GC synaptic transmission was abolished by either blocking NMDA receptors or antagonizing GluN2A subunit-containing NMDA receptors. These results indicate that gestational VPA exposure in mice results in ASD-like behaviors accompanied by increased cerebellar MF-GC synaptic transmission via GluN2A-containning NMDA receptors in the offspring. The results suggest that abnormal sensory stimulation-evoked MF-GC synaptic transmission may contribute to the impaired motor coordination and learning ability seen in ASD model mice.

Although prenatal VPA exposure enhances the risk of ASD by altering synaptic development, transmission, and plasticity [19–26], the neurobiological mechanisms underlying ASD development remain unclear. Cerebellar cortical neurons receive sensory information through the MF-GC pathway and generate commands related to motor coordination and motor learning [50]. A previous study demonstrated deficits in visual-motor and manual dexterity, limb coordination, speed, gait, and coordination in patients with ASD and animal models of ASD [2], which suggests that cerebellar circuit dysfunction may contribute to motor impairment in ASD. Our results show that VPA-exposed mice exhibited deficits in social interaction and increased facial stimulation-induced MF-GC synaptic transmission, suggesting that abnormal transmission of sensory information at cerebellar MF-GC synapses occurs in a mouse model of ASD induced by prenatal exposure to VPA. In addition, abnormalities of cerebellar cortical Crus I/II lobules have been consistently demonstrated in patients with ASD [36, 37]. Previous study demonstrated that ASD model mice exhibit both motor and social deficits, which correlate with a loss of Purkinje cells within the Crus I lobule of the cerebellar cortex [40, 41]. However, the present results showed that gestational VPA exposure produced behaviors of ASD accompanied with an enhancement of MF-GC synaptic transmission in mouse cerebellar Crus II by an increase of GluN2A-contanning NMDA receptor in offspring mice. The present results combined with previous study suggest that ASD produce abnormal neuronal circuit activity in cerebellar Crus I and Crus II.

In the cerebellar cortex, NMDA receptors are found on the membrane of GCs and their axonal boutons, where they play a critical role in modulating MF-GC synaptic transmission and plasticity [28–30, 32, 33, 47, 49]. Facial stimulation-evoked MF-GC synaptic transmission occurs in the absence of GABA receptor activity, which mainly consists of AMPA receptor-mediated fast components, followed by NMDA receptor-mediated slow components. Consistent with a previous study [33], we found that treatment with D-APV abolished the NMDA receptor-mediated slow components of the MF-GC synaptic response, without changing the AMPA receptor-mediated fast components, such as the N1 amplitude. Notably, we found that blocking the NMDA receptor abolished the increase in facial stimulation-evoked MF-GC synaptic transmission observed in VPA-exposed mice, indicating that the increase in MF-GC synaptic transmission seen in this mouse model of ASD is mediated by NMDA receptors. Abnormal NMDA receptor function has been demonstrated previously in several brain regions of VPA-exposed animals [19, 21, 26, 27]. Prenatal exposure to VPA in rats leads to an abnormal increase in NMDA receptor function in the brain, as well as increased expression of GluN2A- and GluN2B-containing NMDA receptors and increased NMDA receptor-mediated synaptic currents in the medial prefrontal cortex [19, 21, 26]. Moreover, pharmacological inhibition of NMDA receptor function in VPA-exposed rats improves their social deficits [24] and rescues their repetitive behaviors [27]. Postnatal administration of a low-dose NMDA receptor antagonist improves ASD-associated behaviors in VPA-exposed rats, indicating that prenatal exposure to VPA induces ASD-like behaviors in adult rats through suppression of NMDA receptor function [27, 46]. Consistent with previous reports [21, 24, 26, 27], our findings indicate that NMDA receptor signaling increases during facial stimulation-evoked MF-GC synaptic transmission in VPA-exposed mice.

GluN2A and GluN2C mRNA are detectable in cerebellar granule cells during the second postnatal week, whereas GluN2B mRNA is transiently expressed in GCs during the first 2 postnatal weeks in rats [31]. GluN2A subunit has been detected in GC somas and parallel fiber boutons in the cerebellar cortex of adult mice [29, 31]. GluN2A subunit- and GluN2C subunit- containing NMDA receptors were detected at the postsynaptic junction in GC dendrites, forming synapses with MF terminals, indicating that GluN2A subunit- and GluN2C subunit- containing NMDA receptors are anatomically concentrated at the MG-GC synapse [32]. However, our previous data have shown that GluN2A subunit-containing NMDA receptor immunoreactivity was expressed in the mouse cerebellar GCs [49], and blockade GluN2A subunit-containing NMDA receptors depressed the facial stimulation-evoked MF-GC synaptic transmission and long-term synaptic plasticity in vivo in mice [33, 47, 49]. The results from the current study show that GluN2A-containing NMDA receptor blockade inhibits MF-GC synaptic transmission and inhibits the increase in facial stimulation-evoked MF-GC synaptic transmission in VPA-exposed mice. These results suggest that the increase in facial stimulation-evoked MF-GC synaptic transmission is mediated by GluN2A-containing NMDA receptors in the cerebellar cortex of VPA-exposed mice. Importantly, GluN2A subunit-containing NMDA receptor expression was at significantly higher levels in the granule cell layer of VPA-exposed mice than in the granule cell layer of control mice, indicating that the increase in facial stimulation-evoked MF-GC synaptic transmission was mediated by GluN2A-containing NMDA receptors in the cerebellar cortex of VPA-exposed mice.

The results from this study show that VPA-exposed mice exhibited increased facial stimulation–evoked cerebellar MF-GC synaptic transmission accompanied by impaired social interaction. The cerebellar GCs are relay neurons, which receive sensory information from mossy fibers and transfer it to Purkinje cell and molecular layer interneurons by high-frequency and fidelity spike firing [51]. Therefore, increasing sensory stimulation-evoked MF-GC synaptic transmission could produce increase the spike-firing activity of GCs and disrupt the high-fidelity transmission of sensory information by GCs, which could impair motor coordination, motor learning, and social interaction. In addition, GluN2A-containing NMDA receptors are overexpressed in the GCs in a VPA-exposed mouse model of ASD, and the increase in sensory stimulation–evoked MF-GC synaptic transmission was abolished by a specific GluN2A-containing NMDA receptor antagonist. These results suggest that GluN2A-containing NMDA receptor–mediated enhancement of facial stimulation-evoked MF-GC synaptic transmission may impair motor coordination, motor learning, and social interaction by disrupting high-fidelity MF-GC synaptic transmission in a VPA-exposed mouse model of ASD. Moreover, cerebellar dysfunction could cause the core ASD symptoms of social communication deficits and stereotyped behaviors. Problems with social cognition could be caused by various factors, such as loss of specific neurons within the motor cortex and cerebellum [34], disruptions in specific cerebro-cerebellar loops [52], and cerebellar signal transfer [27]. The present study showed that increasing facial stimulation-evoked MF-GC synaptic transmission via GluN2A-containing NMDA receptors in VPA-exposed ASD could impair social interaction. Therefore, we speculate that GluN2Acontaining NMDA receptor blockade in GCs may improve ASD-likes behavior in VPA-exposed mice; further research is needed to clarify this point. Collectively, the results from this study improve our understanding of the cellular and synaptic mechanisms of motor impairment in VPA-exposed mice.

## Data Availability

The datasets generated and analyzed during the current study are available from the corresponding author on reasonable request.
